# Text analytics approach to extract course improvement suggestions from students’ feedback

**DOI:** 10.1186/s41039-018-0073-0

**Published:** 2018-06-04

**Authors:** Swapna Gottipati, Venky Shankararaman, Jeff Rongsheng Lin

**Affiliations:** School of Information Systems, Singapore Management University, Singapore, Singapore

**Keywords:** Student feedback, Teaching evaluation, Explicit suggestions, Text analytics, Text mining, Classification techniques

## Abstract

In academic institutions, it is normal practice that at the end of each term, students are required to complete a questionnaire that is designed to gather students’ perceptions of the instructor and their learning experience in the course. Students’ feedback includes numerical answers to Likert scale questions and textual comments to open-ended questions. Within the textual comments given by the students are embedded suggestions. A suggestion can be explicit or implicit. Any suggestion provides useful pointers on how the instructor can further enhance the student learning experience. However, it is tedious to manually go through all the qualitative comments and extract the suggestions. In this paper, we provide an automated solution for extracting the explicit suggestions from the students’ qualitative feedback comments. The implemented solution leverages existing text mining and data visualization techniques. It comprises three stages, namely data pre-processing, explicit suggestions extraction and visualization. We evaluated our solution using student feedback comments from seven undergraduate core courses taught at the School of Information Systems, Singapore Management University. We compared rule-based methods and statistical classifiers for extracting and summarizing the explicit suggestions. Based on our experiments, the decision tree (C5.0) works the best for extracting the suggestions from students’ qualitative feedback.

## Introduction

Universities employ various formal and informal methods to collect and analyse feedback from students in order to enhance the quality of teaching and learning. Many institutions have implemented evaluation surveys which combine “program-wide” questions and “module-specific” questions that enable comparisons to be made across the institution whilst allowing flexibility for individual modules (Keane and Labhrainn 2005; Beran et al. 2007). These surveys provide valuable feedback that helps course designers towards improving teaching style, course content and assessment design and overall student learning (Lewis 2001; Moore and Kuol 2005); Murray 1997). At the same time, the feedback must be analysed and interpreted with great care so that action, and ultimately improvement, can result from feedback process (Lizzio et al. 2002; Beran et al. 2005; Franklin et al. 2001).

Students provide feedback in two distinct forms, namely quantitative (numerical) ratings for questions and qualitative comments related to teaching, content and learning (Hounsell 2003). The teaching component refers to aspects such as instructors’ interaction, delivery style, ability to motivate students and out of class support. The content refers to aspects related to course details such as concepts, lecture notes, labs, exams and projects. The learning refers to aspects related to student learning experience such as understanding concepts, developing skills and applying skills acquired.

**Fig. 1:**
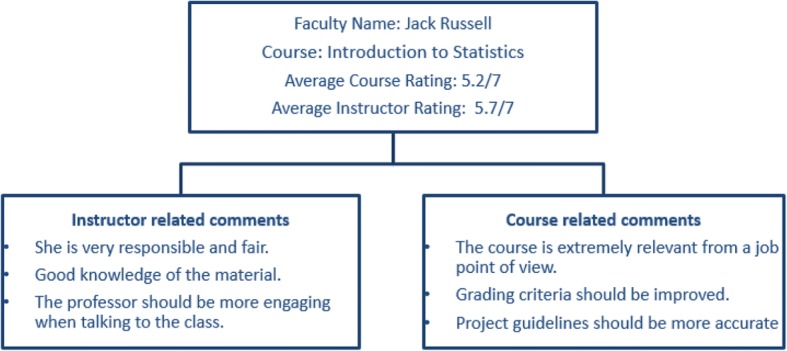
Example students’ feedback for faculty and the course. Both qualitative and quantitative feedback is collected by student evaluation system, FACETS

Singapore Management University (SMU) end-of-term student feedback questionnaire “FACETS” is designed to gather students’ perceptions of the instructor and their learning experience in the course. “FACETS” stands for “For Assessment of Continuing Excellence in Teaching”. The questionnaire was developed in 2012 and it has been used since then. The questions were adapted and developed from the literature on measuring tertiary teaching and learning. The questionnaire is administered online by the Centre for Teaching Excellence (CTE) at the end of every term. The collected data is analysed at individual level, and a summary of the quantitative data as well as compilation of qualitative comments in raw form are made available to the respective instructors as individual reports. Key components of the feedback report are in shown in Fig. 1.

Faculty members are expected to use the feedback in their FACETS reports to identify their strengths and areas for improvement. They are required to reflect on their teaching and curriculum and take steps to improve their instructional strategies and course materials to create a more positive learning experience for future students. More often, an analysis of student feedback falls short of an in-depth exploration of a qualitative feedback (Yao and Grady 2005; Harper and Kuh 2007), thus limiting instructors to the numerical scores and a human understanding of a sample of the feedback, which abstracts collective sentiments for individual components of courses. The question is how to help the faculty to better digest such large amounts of comments and discover the gaps in the course delivery.

Extracting sentiments of students on the course and instructor from qualitative feedback comments and presenting in a user friendly manner is one of the popular approaches adopted by some of the recent works (Altrabsheh et al. 2014; Hajizadeh and Ahmadzadeh 2014; Rashid et al. 2013; Nitin et al. 2015; Shankararaman et al. 2017). In this paper, we particularly focus on extracting suggestions from students’ qualitative feedback comments using text mining approaches. There are several benefits of extracting suggestions from the list of all the feedback comments. Firstly, suggestions provide useful pointers on how to further enhance the student learning experience. For example, the suggestion given by a many students such as “more programming examples should be included”, in a programming course, is an indication that students are not getting enough examples in the course and hence the lecturer can include more examples to enhance student learning. Secondly, when combined with the quantitative feedback, the suggestions help the instructor to prioritize and target the required changes that need to be applied to the course. Usually, the instructor uses the quantitative feedback on questions related to the course and accordingly amends the course for improvements. In addition to using this quantitative feedback, the instructor can use suggestions which most students talk about and amend the course accordingly. For example, if students provided a low score to the question related to “course labs, project and assignment” and then added suggestions in the comment sections, the instructor can combine both these feedback in order to gain a better understanding what needs to be improved. For example, the instructor can analyse where the main concern lies, whether it is in labs or projects or assignments, and amend the course accordingly. Thirdly, suggestions are useful to help improve the instructor’s teaching rating. Through the course evaluation system, the instructor has the opportunity to discover the gaps in teaching delivery and course content. With better insights gained from the student suggestions, the instructor’s overall performance can be further improved. Lastly, the management, dean or associate dean, can use the suggestions, to make decisions with regard to provisioning the necessary training or support to the instructor, for improving teaching delivery and course content.

Suggestions are usually provided in two formats: negative comments and actionable comments. In this paper, we focus on extracting the actionable comments or, in other words, explicit suggestions. One of the main challenges with explicit suggestions extraction task is the textual nature of comments which are expressed in natural language (Stavrianou and Brun 2012). We explain the challenges in detail in “Suggestion extraction task” section. Furthermore, the suggestions are embedded within the text which can consists of facts and sentiments. Opinion mining, topic extraction and natural language processing (NLP) techniques from the text mining and linguistics research are widely popular for mining users’ comments in social media (Liu 2010) and (Sarawagi 2008). Sentiment mining techniques are widely used for product review mining in consumer business world (Hu and Liu 2004). We leverage these techniques for building the solution model for explicit suggestion extraction task. Our solution applies data mining and text mining techniques on qualitative comments to extract explicit suggestions from students’ comments.

The paper will be structured as follows. The “Suggestion extraction task” section describes the suggestion extraction task. The “Literature review” section will be devoted to literature review background on opinion mining, NLP and classification techniques. We describe our research questions in the “Research questions” section. The “Solution model for suggestion extraction” section describes our explicit suggestion extraction solution overview and its details. In the “Data overview and tool implementation” section, we focus on dataset and tool implementation details. The "Results and discussion" section focusses on experiments, results and discussions. We conclude in the “Conclusions” section suggesting some interesting future directions of our work.

## Suggestion extraction task

We will first introduce a few basic concepts of opinion mining.

1. Comment: Qualitative feedback given by a student for a course taken at a university. For example, “The course project is very difficult but very challenging” is a comment for a course code, IS203. The comments can also be multi-sentenced and usually not grammatical in nature as can be seen in the above comment.
2. Opinion: Unlike factual information, opinions are subjective expressions that describe people’s sentiments and feelings towards aspects or entities or events (Liu 2010). For example, “sometimes the instructor talks too fast for us to grasp the concept” is an opinion towards the instructors’ presentation skills.
3. Sentiment: Sentiment refers to the positivity or negativity of a given comment. For example, given the comment, “The course project is very difficult but very challenging”, the sentiment is “negative”. In some applications, a neutral sentiment is also widely used. In our preliminary studies, we observed that the students’ comments are mostly negative or positive.
4. Suggestion: Suggestions refer to comments, which provide actionable feedback to the decision makers such as administrators and faculty members (Jhamtani 2015). For example, “The course needs to focus on the code as much as the business side” is a suggestion from the student feedback on the course content whereas “sounding a little more upbeat may help with the class’s energy level” is a suggestion for instructor.
  Explicit suggestions: Explicit suggestions are expressed as wishes or improvements. (Negi and Buitelaar 2015; Stavrianou and Brun 2012; Brun and Hagege 2013).
  Implicit suggestions: These are similar to the negative opinions. User likes and dislikes are taken into account to make recommendations. For example, in the comment “sometimes the instructor talks too fast for us to grasp the concept”, the implicit suggestion is that “the instructor must slow the pace”.

Usually, the comments are short in nature but they may contain opinions or facts as well as suggestions. For example, the first comment in Table 1, contains an opinion as well as an explicit suggestion. “The course is good” is an opinion and “I do however feel that labs should be done in class to replace ICE” is an explicit suggestion. Also note that the third comment is a negative opinion with context about instructor and can be referred to as an implicit suggestion. In our work, we focus only on extracting the explicit suggestions from the students’ comments. In the next section, we describe the background of opinion mining, NLP and classification techniques popular in extraction or categorization tasks.

**Table 1:** Sample comments from students with sentiments and suggestions

Comment	Sentiment	Implicit suggestion	Explicit suggestion
1. The course is good and I do however feel that *labs should be done in class to replace ICE*	+ive	N	Y
2. Very knowledgeable, patient and easygoing - *sounding a little more upbeat may help with the class’s energy level*	+ve	N	Y
3. Sometime he went through the concepts a bit too fast for us to gasp.	−ve	Y	N
4. Asks challenging questions to get us to think deeper.	+ve	N	N
5. *The course needs to focus on the code as much as the business side.*	None	N	Y
6. *It would be good if the project details are released earlier*.	None	N	Y

## Literature review

In this section, we present the research in the area of opinion mining, natural language processing and classification models. We also focus on the research pertaining to student feedback or teaching evaluations under opinion mining area.

### Opinion mining

**Fig. 2:**
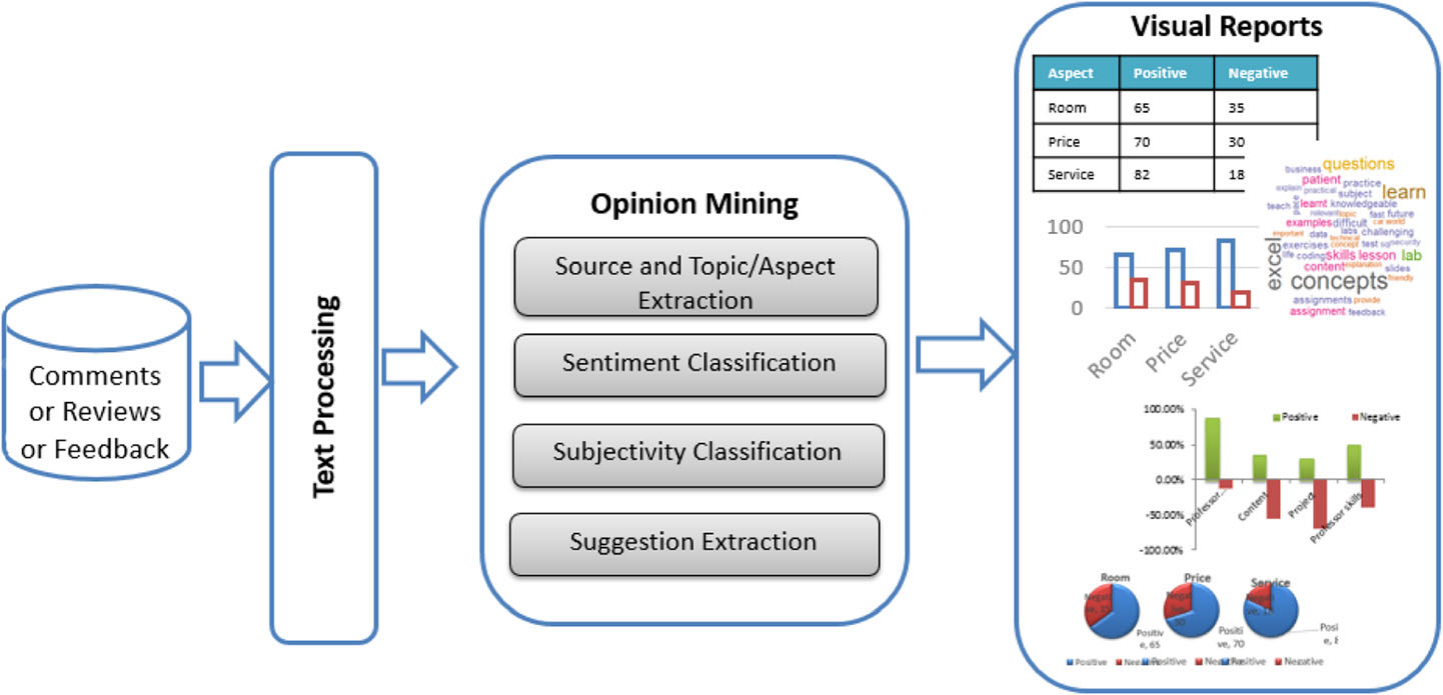
Opinion mining architecture

Opinion mining involves extracting sentiments and feelings from various sources like social media and online forums. Opinions are central to almost all human activities. They are key influencers of our decision-making process. It is a well-studied research topic for the past 10 years mainly focusing on opinion extraction, sentiment classification, opinion summarization and applications in real world (Liu 2010). Its roots can be found in many real-life applications and several application-oriented research studies have been published. Figure 2 shows the general architecture of opinion mining. The users’ comments are taken as inputs to generate sentiment analysis visualizations as outputs that can aid the decision-making process. Summarizing opinions helps organizations such as government and businesses to improve the processes. The details of the opinion mining component is described in the sub-sections.

#### Source and topic extraction

Opinion source or holder is the person or the source who presents the opinion (Liu 2010). The opinion source is important when authenticating the opinion as well as the strength, application and classification of the opinion, as the quality and reliability of an opinion is greatly dependent on the source of that opinion. The opinion topic or the target refers to the person, object, feature, event or topic about which the opinion is expressed. To compare or summarize the comments, it is necessary to automatically identify and extract those topics that are discussed in the feedback. To identify topics at the sentence or document level, the system should be able to identify evaluative expressions (Popescu and Etzioni 2005; Hu and Liu 2004). Moreover, some topics are not explicitly presented, but rather, they are predicted from term semantics, also referred to as implicit features. A background study reveals that the process of opinion topic or target extraction involves various natural language processing tasks and techniques such as pre-processing, tokenization, part-of-speech tagging, noise removal, feature selection and classification.

#### Sentiment analysis

Sentiment classification aims at classifying the data into positive or negative polarities (Pang et al. 2002) using supervised methods or unsupervised methods. Similar to opinion extraction, fine-grained sentiment analysis is desired, as it is highly effective to understand the pulse of the commenters at feature level. The task of sentiment target detection aims at extracting the sentiment targets in the reviews using multiple heuristic techniques (Hu and Liu 2004). Pang et al. (2002) examined several supervised machine learning methods like support vector machine (SVM) and Bayes classification for sentiment classification of movie reviews and showed that classifiers performed poorly on sentences as sentences contains less information (Chang and Lin 2011).

Lexicon methods are based on sentiment words and phrases that are instrumental to sentiment analysis for obvious reasons (Liu 2010). A list of such words and phrases is called a sentiment lexicon (or opinion lexicon). Over the years, researchers have designed numerous algorithms to compile such lexicons: SentiWordNet (Esuli and Sebastiani 2006) and Sentiment lexicon (Hu & Liu, 2004).

#### Suggestion prediction

Unlike opinion mining where we identify the like and dislikes or positive from negative comments, extracting suggestions seeks to discover objective comments or actionable comments indicating what improvement an individual would like to see or have (Stavrianou and Brun 2012). Automatic discovery of suggestions from customer reviews or surveys is vital to understanding and addressing customer concerns. Equipped with this insight, businesses can channel their resources into improving their product or services (Negi and Buitelaar 2015). Our tool extracts suggestions using rule-based and classification approach.

#### Opining mining in education

In this sub-section, we present the works on opinion mining in the context of education. In particular, we present the works on research related to student feedback data.

Student evaluations and opinion mining: In the field of education, Rashid et al. (2013) used generalized sequential pattern mining and association rule mining to analyse opinion words from student feedback. Altrabsheh et al. (2014) use classifier like complement naïve Bayes (CNB) and SVM to learn sentiments from students’ feedback with 84 and 94% accuracy, respectively. Wiebe and Riloff (2005) study pre-labelling methods comparing manual labelling of opinion statements on training data to that of an automated approach in classifying subjectivity. To predict whether a student would retake the course, Hajizadeh and Ahmadzadeh (2014) experimented on student feedback to analyse the sentiments. Yu et al. (2003) retrieved opinions from facts using document similarities approaches such as naïve Bayes and multi-naïve Bayes classifier.

Suggestion prediction: A study by Ramanand et al. (2010) has employed rule-based approach for identifying user wishes. There has been other research works in mining suggestion from sources like, tweets on mobile phone, electronics and hotel reviews. Brun and Hagege (2013) developed a recommender system using customer profile and suggestions. Yang and Fang (2013) demonstrated that suggestion extraction can be applied in user recommendation based on user profile and geographical context. Sapna et al. (2015) extracted suggestions from political datasets. The F-score on political dataset is 70.8%.

In our work, we study the explicit suggestion extraction from the students’ course feedback. To the best of our knowledge, this is the first work in education data analytics research. We use classification-based approaches for extracting explicit suggestions from qualitative comments in our solution model.

### Natural language processing for English

NLP is the research area dedicated in automatic processing of human language. Such processing helps in the subsequent tasks of classification, clustering and opinion mining. Preprocessing the student comment with common natural language processing techniques (NLP) such as stopword removal, parts-of-speech (POS) tagging, lemmatization and bigrams can help increase the accuracy of the suggestion extraction task. In this sub-section, we describe the techniques that are relevant to our solution model.

#### Tokenization

Tokenization deals with the splitting of text into units during data pre-processing. Text can be tokenized into paragraphs, sentences, phrases and single words. The delimiters used in this process vary with data sets.

#### Stopword removal

Stopwords are common English words such as “the”, “am”, and “their” which do not influence the semantics of the review. Removing them can reduce noise. Informative terms like “bug” or “add” will become more influential, which might improve the accuracy of document classifiers. However, some keywords that are commonly defined as stopwords can be relevant for the review classification. For instance, the terms “should” and “must” might indicate a feature request, “did”, “while” and “because” a feature description, “but”, “before” and “now” a bug report and “very”, “too”, “up” and “down” a rating.

#### POS tagging

POS tagging focuses on reading in a text and assigning parts of speech to a word. For the tagging of English language text, the Penn Treebank tag set is used in annotating tags to words (Marcus et al. 1993). By tagging parts of speech to a paragraph of text, we can identify the relevant groups of words that form up the entities within a paragraph of text. The most common entities are person names, locations and organizations.

### Classification techniques for textual data

In this section, we introduce various commonly used classification techniques that can automatically classify the comment type. One of the goals of text mining is to classify documents into predefined categories. Training a machine is also known as supervised learning where an instance of a set of documents with pre-defined human-labelled categories are used for training. Supervised learning algorithm study features within the document and corresponding classes or category. A model is then used to test on a new set of document and produce an estimate of the category it falls into.

Unsupervised learning method is another approach to document classification. Unlike supervised learning, it does not require machine to learn from a set of human-labelled documents but instead sort to split the feature within a document based on criteria or rules. Previous studies employ the use of rule-based method that detects modal verbs or phrase pattern (Ramanand et al. 2010; Negi and Buitelaar 2015). We describe both the models in the following sub-sections.

#### Rule-based classifier

The most trivial technique to automatically categorize a student comment is to check whether it contains a certain keyword. We can manually define (and possibly maintain) a list of keywords that we expect to find in a comment, a negative feedback or a positive feedback or a suggestion (Brun and Hagege 2013). We then check whether one of the keywords is included in the text. For this, we can use regular expressions in tools like grep, string matching libraries or SQL queries, while ignoring letter cases and wrapping around the keywords (e.g. using “LIKE” in SQL or \p in grep).

For suggestion extraction, we propose a rule-based approach similar to Negi and Buitelaar (2015) and applied it on student comments. A sentence will be categorized as a suggestion if it follows one of these rules.

1. Pattern matching: Phrase that matches with “should”, “could”, “include”, “could have” or some with similar intent phrases are indicators of suggestions. We came up with a list of phrases, a thesaurus as shown in Table 2 through empirically observing students’ comments, similar to Brun and Hagege (2013).
2. POS tagged: Modal verbs (MD) are followed by a verb (VB, VBZ, VBP). The task of the speech tagging is performed using NLTK (Bird et al. 2009).
3. POS tagged extended: Tag list includes noun plural (NNS) and proper noun singular (NNP) as described by Marcus et al. (1993).

**Table 2:** Sample text phrases commonly used in expressing suggestion

Suggestion phrases	
should have, have more, suggestion, perhaps, could be, can be, could give, could provide, could explore, better if, etc.,	

#### Decision tree classifier (C50)

**Fig. 3:**
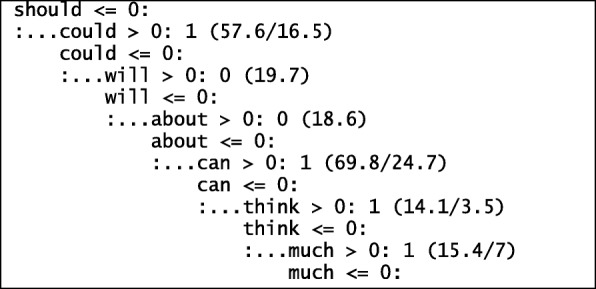
Feature of importance. For example, “should” is an important predictor in this tree

C50, also known as decision trees (DT) algorithm (Quinlan 1993; Kuhn et al. 2015) is a statistical classifier. It seeks to split or divide features from a document to classes or category. The root node normally gives the best prediction compared to those down the tree. A snippet of the trained model on student suggestions is shown in Fig. 3. C50 comes with tuning parameters such as number of trials, model type and feature selection. We can specify the number of boosting iteration, choose a tree or rule-based model and whether to include feature selection for our model.

#### Support vector machine (SVM)

SVM algorithm finds a hyperplane that demarcates the classes or categories by their features over a space (Cortes and Vapnik 1995). It seeks to maximize the distance between the planes and points that falls on the edge of the plane which are known as support vectors. A key concept required for defining a linear classifier is the “dot” product between two vectors, also referred to as an inner product or scalar product.

#### Conditional inference trees (Ctree)

**Fig. 4:**
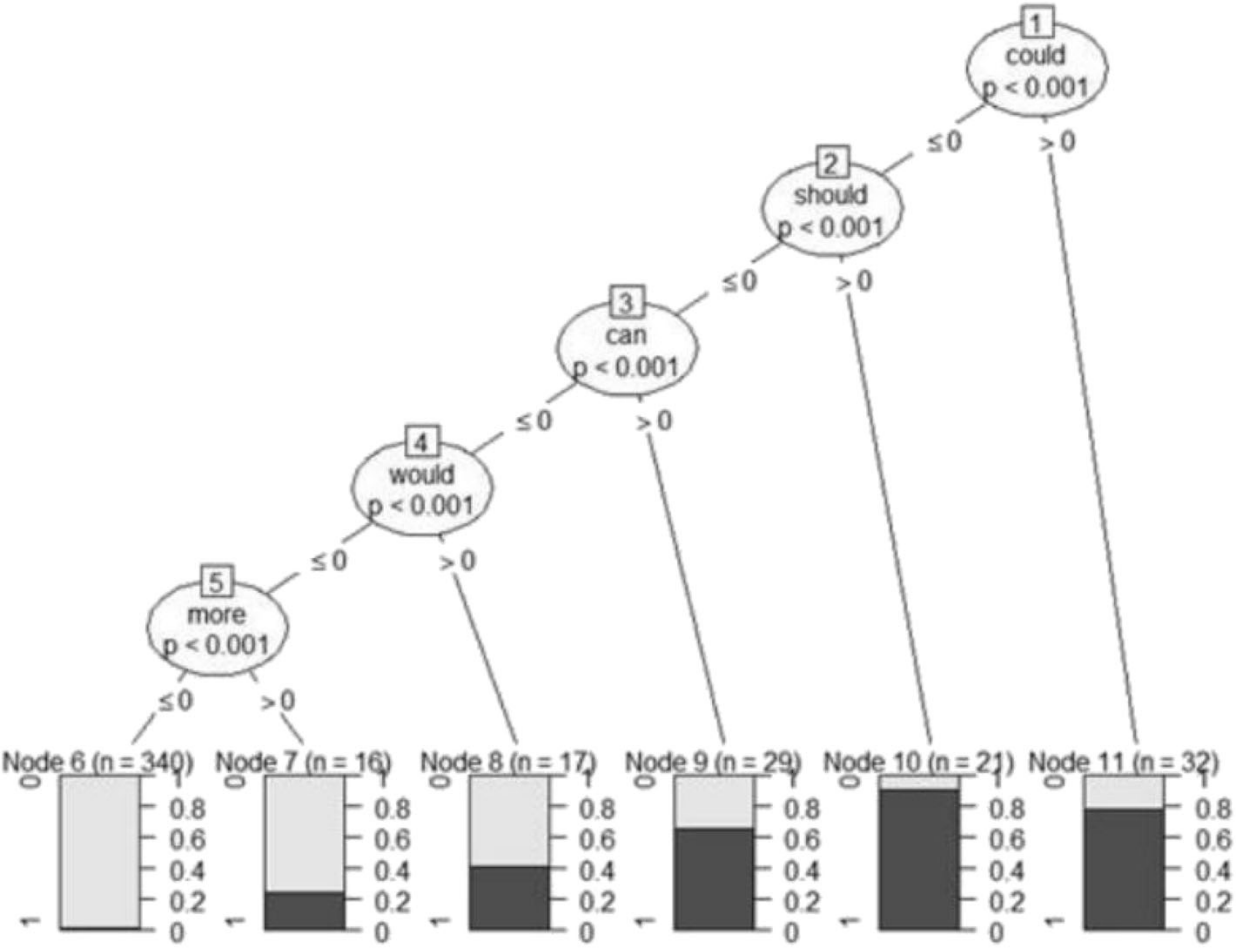
Ctreemodel plot for student comments that are suggestions

Conditional inference trees work much like C50 decision trees. However, it uses significance test procedures to select variable and maximizing information measures (Hornik 2016; Hothorn et al. 2006; Hothorn et al. 2016). Figure 4 shows a model plot of Ctree. Variables such as “could” and “should” have low *p* value and hence maximizes the performance of the classifier.

#### General linear model (GLM)

GLM works on a fundamental principle of linear regression, line fitting (Madsen and Thyregod 2011; Hastie and Pregibon 1992). Each predictor has a coefficient with an assigned level of significance or correlation to a certain class as shown in Table 3. The asterisk indicates significant predictors. Words like “can”, “could”, “should”, and “would” have great significance with low *p* value and a positive coefficient.

**Table 3:** Linear model outcomes on sample student comments dataset

Word	Estimate	Std. Error	*z* value	Pr(>|*z*|)
able	−13.916	4316.421	−0.003	0.997
can^*^	8.959	1.965	4.559	0.000
could^*^	6.967	1.261	5.526	0.000
have	3.217	1.370	2.348	0.019
its	−3.850	3.248	−1.185	0.236
just	3.766	1.849	2.037	0.042
like	−14.567	4063.761	−0.004	0.997
little	−2.916	1.273	−2.292	0.022
should^*^	8.808	1.894	4.651	0.000
taught	3.873	1.635	2.368	0.018
would^*^	4.301	1.211	3.552	0.000

*indicate the significant predictors

## Research questions

In this section, we summarize the research questions for our project. Firstly, our goal is to study how to combine opinion mining and NLP research to derive a solution model for the suggestion extraction task. Secondly, we study how accurately the classification techniques from “Literature review” section can predict the comment type. This includes answering the following questions:

- RQ1—Solution model: How should the comments metadata, text classification, NLP and sentiment analysis be combined in order to classify the suggestions? (“Solution model for suggestion extraction” section)
- RQ2—Rule-based models: Which rule-based model performance better (pattern matching, POS tagged or POS-tagged extended) in extracting suggestions from the comments? (“Rule-based method results and analysis” section)
- RQ3—Classification algorithms: Which classifier algorithm performance better (decision tree, vs SVM vs GLM vs Ctree) in extracting suggestions? (“Statistical classifier results and analysis” section)
- RQ4—NLP techniques: What is the impact of stopwords on the accuracy of the classifications? (“Statistical classifier results and analysis” section)

## Solution model for suggestion extraction

In this section, we first present the overview of our solution and then the details of each component of the solution.

### Solution model overview

**Fig. 5:**
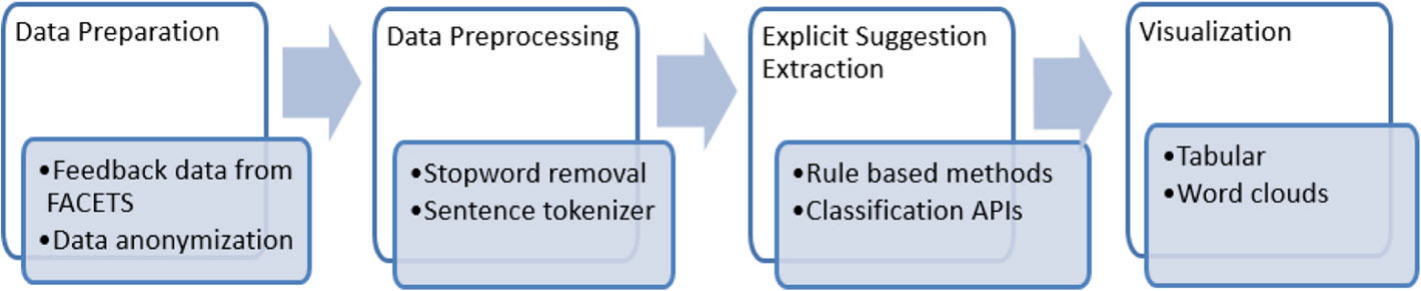
Solution model for suggestion extraction task

Figure 5 shows the overview of our solution model for explicit suggestion extraction. The solution approach consists of three main stages. In the first stage, raw input comments are anonymized, pre-processed and prepared for suggestion extraction stage. The second stage is critical to our solution approach. This stage employs text mining algorithms for the extraction of suggestions from the processed comments. In the final stage, the extracted suggestions are aggregated for comprehensive reporting that can used by the instructors and administrators of improving the teaching and learning process.

### Solution model details

Recollect that FACETS tool consists of both quantitative and qualitative survey questions. The qualitative data is derived from the two open-ended questions about course and instructor. The input for our solution approach is the qualitative data from all courses in the University. In the first stage, we collect the data and anonymize the data. The data consists of faculty names, course names and course ID’s which are very sensitive confidential information. Hence, the faculty names and course names are anonymized.

In the second stage, to pre-process the data, individual sentences are extracted from input comments using sentence tokenizers. Tokenization deals with the splitting of text into units during data pre-processing which is critical for the second-stage algorithms. We also adopt a vector space representation of a document where each comment is evaluated as document term frequency (Manning et al. 2008). Further, we implement stopword removal API in the data cleaning process.

The third stage involves extracting explicit suggestions using text classification methods. In our experiments, we used four different classification algorithms described in the “Literature review” section. We also implement rule-based methods to extract the suggestions from comments. In our experiments, we evaluate these techniques on the accuracy of extracting suggestions from all the comments.

In the final stage, the goal is to provide user-friendly summaries of the suggestions obtained from student comments. The design goal is to ensure a user-friendly visualization interface that supports search, comparison and analysis. A graphical representation of the text using a word cloud, which is a popular approach, is adopted to provide a quick view. Additionally, we also designed query-based table style suggestions for better usability. We depict a sample screen from the dashboard in the “Visualizations” section.

In this section, we answered our first research question, RQ1, where we proposed a solution model by combining various techniques from opinion mining, NLP and classification APIs. In the subsequent sections, we describe datasets and experiments to evaluate our solution model.

## Data overview and tool implementation

### Data collection and processing

The dataset is the qualitative feedback comments submitted by students attending undergraduate core courses offered by the School of Information Systems at Singapore Management University for two terms in a year. Not all comments are useful for analysing. For example, comments such as “NA” and “Nil” are discarded as they introduce noise into the datasets. After cleanup, we have a total of 5342 comments for our experiments.

Data preparation for experiments: To evaluate various classification methods, we first randomly sampled a small dataset, then we manually labelled the comments that are suggestions and, finally, tested various classification methods described in the “Classification techniques for textual data” section. To compare the models, we used text evaluation measures: precision, recall and F-score (Manning et al. 2008). Precision is the fraction of comments that are actually suggestions among the total number comments classified as suggestions. Recall is the fraction of actual suggestions that have been retrieved over a total number of suggestions in all the student comments. F-score is the harmonic mean of precision and recall.

We used a random subset of 399 comments to perform training and testing. We first perform sentence tokenizing on each of the 399 comments, which produced 604 sentences. This sentence-level approach is useful because comments could contain a mix of subjective and objective sentences. Two example sentences are shown below.

1. “Flexibility in coming up with our own scenarios is great so that we are not entirely restricted. The release of the second project could be earlier so that the timeline for completing it will be less rushed.”
2. “Enthusiastic and entertaining. Classes were never boring. More in class exercises would be good to have.”

In sentence (1), the student first expresses a positive comment regarding project scenario and later provides a suggestion. Sentence (2) shows a couple of subjective phrases followed by a suggestion, “more in-class exercises”. Sentence (2) is tokenized into three sentences in order to isolate the suggestion provided. Table 4 gives details of the datasets. The details of the training and testing data preparation will be described in the next section.

**Table 4:** Datasets for training and testing

Dataset	Raw data	Sentence tokenize	Noise filter
Training and testing set	399	604	568
Full data set	5342	7823	6308

### Data labelling

To train and evaluate our solution model, the first task is to label the data by a human. The human is requested to label the data as follows.

1. If the sentence is a positive sentiment, the label given is “P”.
2. If the sentence is a negative sentiment, the label given is “N”.
3. If the sentence is a suggestion, the label given is “S”.
4. If the sentence is either a fact or none of the above, the label given is “O”.

Out of 568 sentences, 17.25% of the sentences are manually annotated as “S”, suggestions. We used 80–2s0 distribution for training and testing our solution model.

### Tool development

The tool was built on Django, a python-based web framework and is known for its scalability. The web tool supports multiple users, database access and an authentication protocol. Therefore, a secure authentication system is necessary to manage SMU’s faculty data. We setup Django with user authentication and it conveniently comes with an administrator access. While we use python to run the suggestion extraction analysis, the presentation layer is built based on JSON structure. This ensures high performance of the server even when accessed by multiple users. We use D3, which is a javascript-based library for visualizing our data. D3 creates interactive charts or graphs from JSON structured data. The D3 scripts are incorporated into html for web application presentation.

## Results and discussions

In this section, we describe various experiments to answer our research questions, RQ2, RQ3 and RQ4.

### Rule-based method results and analysis

Rule-based experiments answer research question, RQ2 (Table 5). We evaluated all three rule-based methods described in the “Suggestion extraction task” section.

**Table 5:** Rule-based classification. High F-Score indicates good performance

No.	Methods	Precision	Recall	F-score
1	Rule-based (Pattern matching—Rule 1)	0.598	0.598	0.598
2	Rule-based (POS tagged—Rule 2)	0.551	0.793	**0.650**
3	Rule-based (POS tagged extended—Rule 3)	0.340	0.890	0.492

We notice that the first rule approach of extracting exact matching phrase like “would be” or “can be” is easiest to implement but has some drawbacks. For example, for the given sentences, the first rule is unable to identify the pattern since it is not included in the list.

1. “Can *work* on articulating himself better, but nevertheless knowledgeable”
2. “Can *provide* more feedback with regards to the project.”
3. “Could *include* more information on what are the project requirements.”

There is a large verb variation of the modal words in English language and building a huge phrase pattern will be both tedious and costly.

Recall that in rule 2, we included part of the speech tagging on each comment. Hence, any modal verb tagged with MD follow by a verb form like third person singular present (VBZ) or non-third person singular present (VBP) will be classified as a suggestion. However, we noticed that there are other structures in the tag list of comments that are suggestions such as (1), (2), (3) and (4) which were misclassified.

1. “Felt that this should not have been a compulsory module.”
  VBD IN DT *MD RB* VB VBN DT NN NN
2. “Assignment 2 grouping should not be randomized.”
  JJ CD VBG *MD RB* VB VBN
3. “More bridging between theory and practice.”
  *RBR NN* IN NN CC NN
4. “Could include more information on what are the project requirements.”
  *NNP VB* JJR NN IN WDT VBP DT NN NNS

Although extending the rules (Rule 3) to extracting noun plural (NNS) and proper noun singular (NNP) gives a higher recall, it lacks precision. Example comments such as (1) and (2) are misclassified.

1. “Content covered in her lectures are doable and within scope”
  *NN VBD* IN PRP NNS VBP JJ CC IN NN
2. “Always open to student’s views and supportive of them.”
  *NNS VBP* TO NN POS NNS CC NN IN PRP

These experiments answer the research question RQ2; rule-based POS tagging (rule 2) provides higher F-score compared to other rules. Furthermore, from our results, both rules 2 and 3 have high false-positive misclassification. Additionally, rule-based POS tag extraction can also provide automated labelling when human labelling comes at a cost and time (Wiebe and Riloff 2005). To further improve the accuracy of the rule-based tagging, more phrases should be added to the list, and it would be tedious to build a large library of phrases and support a stringent pattern extraction.

### Statistical classifier results and analysis

In this section, we first present the stopword usage experiments to answer research question, RQ4 followed by statistical classifier experiments to answer research question, RQ3. Table 6 shows the results of F-score on all classifiers for stopword experiments.

**Table 6:** F-score showing with and without stopwords. Both indicates the high performance

Stopwords	GLM	SVM	Ctree	C50
With	0. 658	0.735	0.698	**0.781**
Without	0.299	0.477	0.286	0.182

We observe that removing stopwords lowers the performance of the classifier. Most frequently used words in English such as “be”, “has”, “if”, “and”, and “on”, carry no information, and therefore, removal of stopwords is a common technique to improve performance. However, from our experiments, removal of such functional words would result in the loss of vital features like “should”, “more”, “could”, “would” and “have” and this leads to inaccuracy, as shown in Table 6. To answer RQ4, the stopwords are essential for the suggestion extraction task.

We then evaluated four statistical classifiers described in the “Suggestion extraction task” section and observed that SVM and decision tree (C5.0) give a consistent performance in their F-score as shown in Table 7. We observed that SVM and C5.0 give high precision and recall scores. C5.0 gives higher F-score of 78.1%.

**Table 7:** Evaluation results using different classification methods

Classifier	Precision	Recall	F-score
Generalized linear models (GLM)	0.676	0.650	0.658
Support vector machine (SVM)	0.755	0.719	0.735
Conditional inference tree (Ctree)	0.781	0.681	0.698
Decision tree (C5.0)	**0.802**	**0.775**	**0.781**

Bolded scores indicates top performance

We further manually analysed the results to study the misclassifications. Table 8 shows some example comments and the predicted values by C5.0 classifier. Actual represents the labelling by humans and predicted is the machine outcomes. We observed that the misclassified comments by the machine tend to have poor grammatical structure. One possible way of improving the tool performance is combining the rule-based or pattern-based techniques.

**Table 8:** Sample comments from the dataset and the predictions by the tool as suggestions (Yes) or not (No). Bolded are incorrectly predicted comments

Comments (sentence tokenized)	Actual	Predicted
“Prof could have given more leeway to teams seeking to enhance automation for clients.”	Yes	Yes
“We should have more practices in class to allow us to learn more stuff.”	Yes	Yes
“Lessons can be more engaging, by asking the students questions or trying out models.”	Yes	Yes
“Course could have spent more time on app logger and less time on the rest of the stuff.”	Yes	Yes
**“He tries to make the lessons as structured as he can.”**	**No**	**Yes**
**“Prior to this course, I never knew that Excel could be used to analyse or project future sales.”**	**No**	**Yes**
**“Probably organize lab sessions once a week for students to clear their doubts when they are using excel.”**	**Yes**	**No**
“Spends more time going through the examples as some students take more time to understand.”	No	No

### Visualizations

**Fig. 6:**
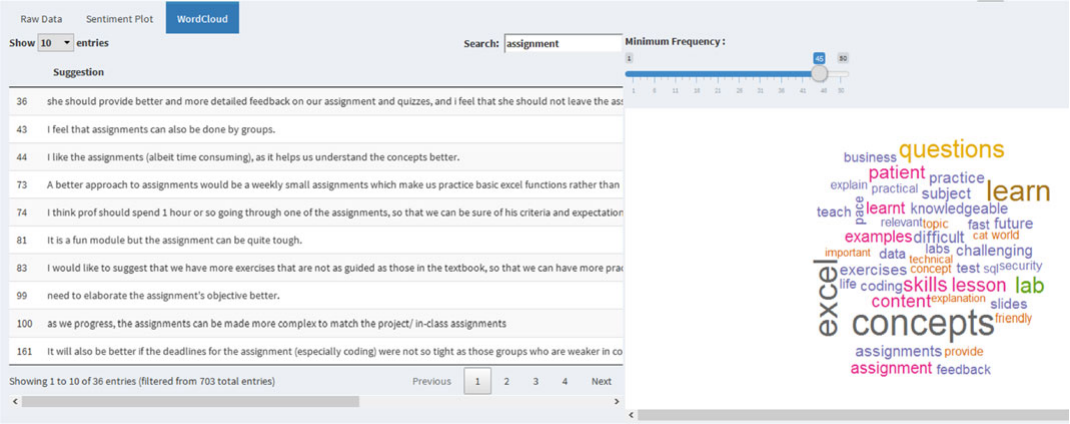
Suggestions table and word clouds on the comments. The tool enables to study a single course or compare across different courses

For reporting, we use the tool *Shiny* (Chang 2016; Fellows 2012) to build a web application. To the left of Fig. 6, the suggestions are presented in a tabular format and on the right is a word cloud (Ian, 2014). The word cloud gives an aerial perspective of the suggestion data, words that are of importance are highlighted by their size and color. User can use the word clouds as a reference to further refine their search. For example, if a user would like to know what suggestion is given for the word “assignment” which is highlighted in pink, the user can enter a search entry on the left.

In the example shown in Fig. 6, we observe that students provide a number of suggestions relating to the word “assignment” for topic like projects. They include “assignment to be done in groups”, “provide clear objectives or direction” and “assignments to be in chuck size”.

### Discussions

The current research in student feedback is majorly dedicated in sentiment extraction. Various techniques were proposed to detect positive and negative opinions from the comments. However, the students also tend to provide suggestions to the instructor and extracting such suggestions will aid the instructor to improve the course design and delivery. Though negative comments can be treated as suggestions, students tend to provide explicit suggestions which are usually tagged as neutral by the existing algorithms and techniques. Our project fills this gap by providing techniques to extract suggestions from students’ teaching evaluations. Suggestions in a way provide ideas for the instructor on how to improve the course. Automated suggestion extraction from students’ comments aids instructors to quickly focus on those that are actionable. The instructors may change their teaching style or course content based on these actionable suggestions. We proposed two solution approaches for the suggestion extraction task. The first approach is rule-based methods. From our experiments, we observe that rule-based POS tagging method provides 65% higher F-score compared to other rule-based methods. To further improve the accuracy of the rule-based tagging, more phrases should be added to the list. However, it would be tedious to build a large library of phrases and support a stringent pattern extraction. Our second proposed approach is based on classification models. We observed that both classification models, SVM and C5.0, provide high accuracy in extracting the suggestions compared to other methods. In particular, we also observe that C5.0 gives a higher F-score of 78.1% and is the better model for the suggestion extraction task. In the next section, we present the conclusions and interesting future work.

## Conclusions

In this paper, we proposed a solution model for explicit suggestion extraction from student feedback comments. We evaluated rule-based methods and statistical classifiers for extracting and summarizing suggestions in the domain of education. While rule-based method is a straightforward approach in detecting suggestion through a pattern of clues, as shown from our experiments, it can be a challenge to detect suggestions that do not conform to the rules. The need to expand the rules can be tedious and time-consuming.

Compared to rule-based methods, the support vector machine and decision tree (C5.0) provide high overall classification performance. Additionally, we found that the decision tree C5.0 classifier provides better performance with F-score of 78.1%. We also evaluated the classifier on stopwords experiments and results indicate a lower F-score on stopword removal scenario. Thus, overall, C5.0 works the best for this problem domain.

Our future works includes extracting the topics within a suggestion; this would provide specific insights on what are the areas of improvement and highlight the main concern within the suggestion. Based on feedback from the instructors, we are working on further refining the visualization aspect of the dashboard. For example, we intend to include a bar chart comparison of the number of suggestions for various aspects of the course and also display the frequency of each suggestion. Studying the impact of this research in other schools and other faculties is an interesting future work. Another interesting related future work in this area of students’ feedback or class room participation is in-class settings. Students participate in several activities in the classroom but capturing the students’ emotions or the audio feedback in the class will enable the faculty to intervene the classroom delivery and accommodate the student needs for better learning experience. The new classrooms are equipped with the videos and at the same time other technology aspects such as wireless networks, Wi-Fi settings, and mobility. Capturing the students’ emotions and feedback in-class and discovering insights using text analytics approach will provide timely inputs to the faculty to improve the teaching process.

## Abbreviations

C5.0: Decision tree
CNB: Complement naïve Bayes
CTE: Centre for Teaching Excellence
FACETS: For Assessment of Continuing Excellence in Teaching
NLP: Natural language processing
POS: Parts of speech
SMU: Singapore Management University
SVM: Support vector machine
